# Supplementary figures and images for: Microbial metabolite deoxycholic acid shapes microbiota against Campylobacter jejuni chicken colonization
Source: PLoS One. 2019 Jul 5;14(7):e0214705. doi: 10.1371/journal.pone.0214705 (PMC6611565; doi:10.1371/journal.pone.0214705)

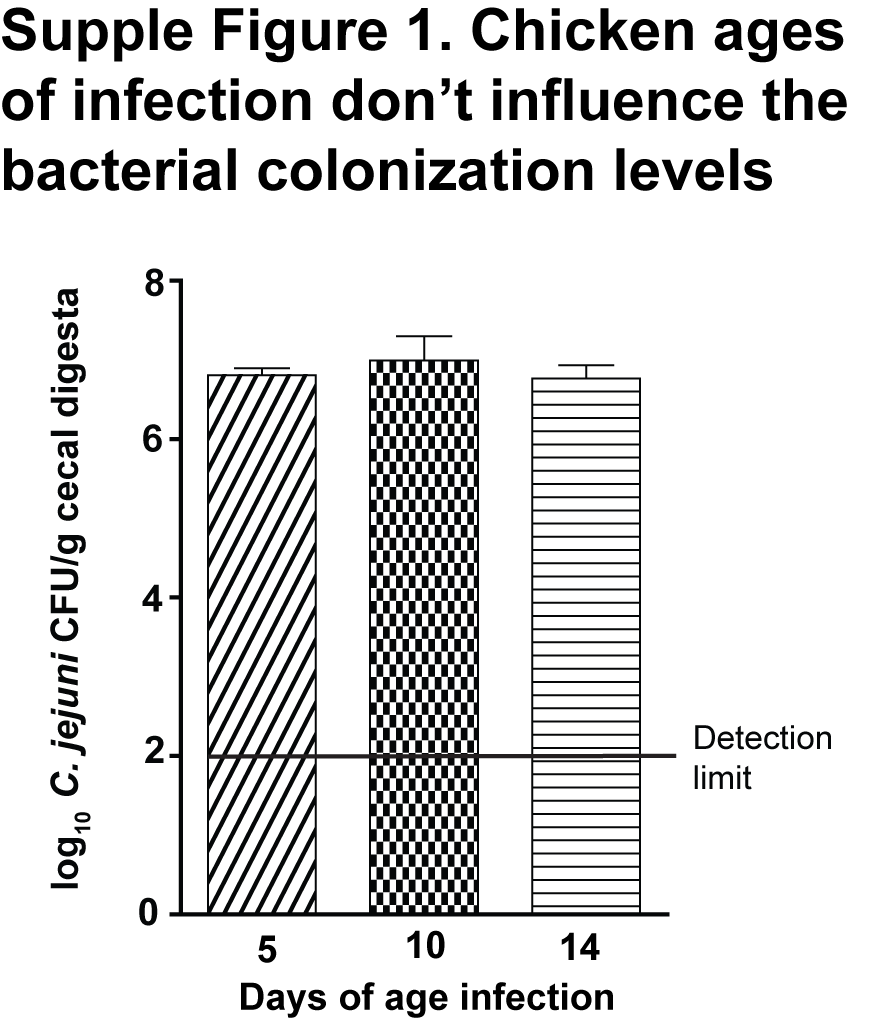

Supplement: S1 Fig — Cohorts of 18 one-day-old broiler chicks were fed basal diet and orally gavaged with 109 CFU/bird C. jejuni AR 101 at 5, 10, or 14 days of age. At d 28, birds were humanely sacrificed and cecal samples were collected. Cecal digesta samples were serially diluted and cultured on Campylobacter selective media. Colonies were enumerated and C. jejuni colonization levels were calculated. All graphs depict mean ± SEM. Results are representative of 2 independent experiments. (TIF) [file pone.0214705.s001.tif]
